# Male food defence as a by-product of intersexual cooperation in a non-human primate

**DOI:** 10.1038/srep35800

**Published:** 2016-10-24

**Authors:** T. Jean M. Arseneau-Robar, Eliane Müller, Anouk L. Taucher, Carel P. van Schaik, Erik P. Willems

**Affiliations:** 1 Anthropological Institute and Museum, University of Zurich, Winterthurerstrasse 190, Zurich 8057, Switzerland; 2 Inkawu Vervet Project, Mawana Game Reserve, KwaZulu Natal, South Africa

## Abstract

Males in a number of group-living species fight in intergroup conflicts to defend access to food resources, a seemingly paradoxical behaviour, given that this resource does not usually limit male fitness directly. We investigated the mechanism(s) driving apparent male food defence in wild vervet monkeys (*Chlorocebus aethiops pygerythrus*) by testing the effect that female resource access, and female audience size and activity had on the response of focal males during simulated intergroup encounters. Males do not appear to defend food to increase the reproductive success of female group members because their response was not influenced by the presence of provisioning boxes that only females could access. Female audience size was also unimportant, suggesting males do not participate in intergroup encounters to advertise their quality to potential mates. However, focal males almost always followed/supported female group members who initiated an approach towards simulated intruders, supporting that male participation largely functions to gain status as a cooperative group member, and that apparent male food defence in this species arises as a by-product of intersexual cooperation. Our study highlights that considering audience composition and activity can reveal the presence of social incentives and illuminate the evolutionary mechanism(s) promoting joint action in intergroup aggression.

Although groups of many social species engage in aggressive intergroup conflicts with their neighbours^1^,^2^,^3^,^4^,^5^, in a number of species (including many primates), males are either the only sex that fights, or the sex that participates most actively^6^,^7^,^8^,^9^,^10^. In humans, males may gain both resource based and immaterial benefits from participating in intergroup conflicts (i.e. warfare)^11^,^12^. Resource-based benefits are obtained from seizing valuable items from neighbouring groups, including food, mates and territory; conversely, immaterial benefits are social incentives that group members bestow on warriors^12^. For example, men who participate in intergroup conflicts may improve their status with group members, and as a result enjoy increased access to allies or wives^11^,^12^. There is little evidence, however, that non-human animals use such social incentives to promote participation in intergroup conflicts, or in any other cooperative activity^13^,^14^.

In many non-human animals, males fight in intergroup conflicts to protect their offspring or defend access to mates^3^,^15^,^16^,^17^, both of which could be considered resource-based benefits. There is also evidence for male food defence in some species^9^,^18^,^19^,^20^,^21^, but given that male fitness is primarily limited by access to receptive females^22^ rather than food resources, this behaviour is seemingly surprising. It is possible that males defend access to food resources to enhance the reproductive output of females and/or the survival of their offspring^9^,^18^, or to attract more mates (resource defence polygyny^23^). Alternatively, male food defence may simply arise as a by-product when males fight for other resource-based benefits^24^ or to gain social incentives. For example, while protecting their mates from harassment by extra-group males, males may end up defending food resources (the ‘Hired Guns’ hypothesis^25^). Potential social incentives gained from food defence include social prestige, or building status as a cooperative group member. According to the social prestige hypothesis, males participate in high-risk and energetically costly intergroup conflicts to signal their genetic quality to potential mates^26^; females who choose to mate with the best fighters ensure their offspring will have these superior genes. The status building hypothesis proposes that because range defence is often a joint action, participation in intergroup conflicts is typically a cooperative activity; therefore, males may participate in intergroup conflicts to enhance their status as a ‘good cooperative partner’. Given that food defence improves access to the resources that limit female fitness, doing so is most likely to enhance status with female group members rather than with males. If this is the case, apparent male food defence may be driven by mechanisms that promote intersexual cooperation (e.g. direct and/or indirect reciprocity mechanisms^27^,^28^,^29^,^30^). For example, a number of studies have found evidence that direct reciprocity can promote cooperative behaviour in primates, and that various services are often traded (e.g. tolerance, grooming, support in agonistic interactions, food sharing and sex^31^,^32^,^33^,^34^,^35^,^36^,^37^). It is possible that defending resources that limit female fitness is a service that males trade with females. In return, females may also provide a variety of services, but when females are able to exert choice in mating partners, sex is the most valuable commodity with obvious fitness benefits for males who are preferred partners.

A recent study on intergroup aggression in vervet monkeys (*Chlorocebus aethiops pygerythrus*) found apparent evidence for male food defence^19^, but the mechanism(s) that drive this behaviour are unclear. As noted above, male food defence possibly serves to increase female reproductive success, to gain social prestige, to build status as a cooperative group member, or males may simply be acting as ‘Hired Guns’. All these functions are possible in vervet monkeys and the four mechanisms are not mutually exclusive. Vervet monkeys live in multi-male, multi-female groups in which females are philopatric and males disperse multiple times throughout their lives, starting at sexual maturity^38^. Parallel dispersal of males does occur^38^, but it is not common. Therefore, adult males are unrelated to their female group members, and also usually unrelated to each other. Males are approximately 1.5 times larger than females, yet both sexes participate actively in intergroup conflicts^19^. Females often initiate intergroup aggression and males frequently support them when they do so^19^. Both sexes direct aggression at both male and female members of opposing groups^19^,^39^. Because the ‘Hired Guns’ hypothesis predicts that food defence arises only as a by-product of male-male competition over mates, and male intergroup aggression is often targeted at females^19^,^40^, the ‘Hired Guns’ function is unlikely in this species. However, larger vervet groups tend to have higher-quality home ranges and experience slightly higher infant survival^41^, indicating that greater resource availability could potentially increase female reproductive success. Female vervet monkeys are able to refuse matings (i.e. exert mate choice) and are highly promiscuous, mating with multiple male group members^19^,^42^. Mating skew is low with two or three males typically gaining a high proportion of matings each year, and male mating success correlates with their propensity to participate in intergroup conflicts^19^. As such, apparent male food defence could be a by-product, produced as males signal their genetic quality to female group members. Alternatively, this correlation could equally mean that apparent male food defence is a by-product of mechanisms that promote intersexual cooperation, for example, that males trade support in intergroup conflicts for sex. Recent experimental work in vervet monkeys demonstrates that females trade grooming for tolerance and support in intragroup conflicts^37^, indicating that a reciprocity-type mechanism could potentially function to promote intersexual cooperation as well.

To elucidate which mechanism(s) underlie apparent male food defence in a wild population of vervet monkeys, we took an experimental approach in which we used playbacks to simulate the presence of a neighbouring group nearby when females had, or did not have, access to provisioning boxes (i.e. a high-quality food resource). Provisioning boxes contained slices of apple with two pieces of corn imbedded in them, and were operated by observers such that access was restricted to female group members. During all playback trials we recorded the response of focal males, as well as the composition of their audience and the response of audience members to the simulated intruders. If males defend food resources to increase female reproductive success, then males should exhibit the strongest response to simulated intruders when females had access to high-quality resources, and their response should be independent of the composition and/or response of audience members. If males participate in intergroup conflicts to gain social prestige, then the size of the female audience (i.e. number of potential future mates) present to observe the honest and costly signal should have the strongest influence on the response of males. Conversely, if apparent food defence is a by-product of intersexual cooperation, then the response of males should be most influenced by the presence of a cooperative partner. Therefore, if a female group member instigates an approach towards the simulated intruders, then focal males should follow and support them.

## Results

We conducted a total of 24 playback experiments in three wild groups of vervet monkeys. During playback experiments, the presence of an intruding group was simulated and the response of one to three focal males was recorded. In 12 of these playback trials, females had access to provisioning boxes that were a source of high-quality food resources, and in the remaining 12 trials, no provisioning boxes were present. Focal males had previously been trained to learn that provisioning boxes could only be accessed by female group members, and therefore, that they would personally gain no resource-based benefit from defending them. Males typically responded to the playback stimulus by orienting their bodies towards the speaker and so we did not include any trials in which the focal male did not acknowledge the playback stimulus in this manner in our analyses. Males usually remained vigilant towards the simulated intruders for 5 to 10 seconds before beginning to monitor the behaviour of their group members. In some cases, males began to approach the speaker soon after checking on their group members, while in other cases they monitored the behaviour of their group members for many minutes before approaching.

**Figure 1:**
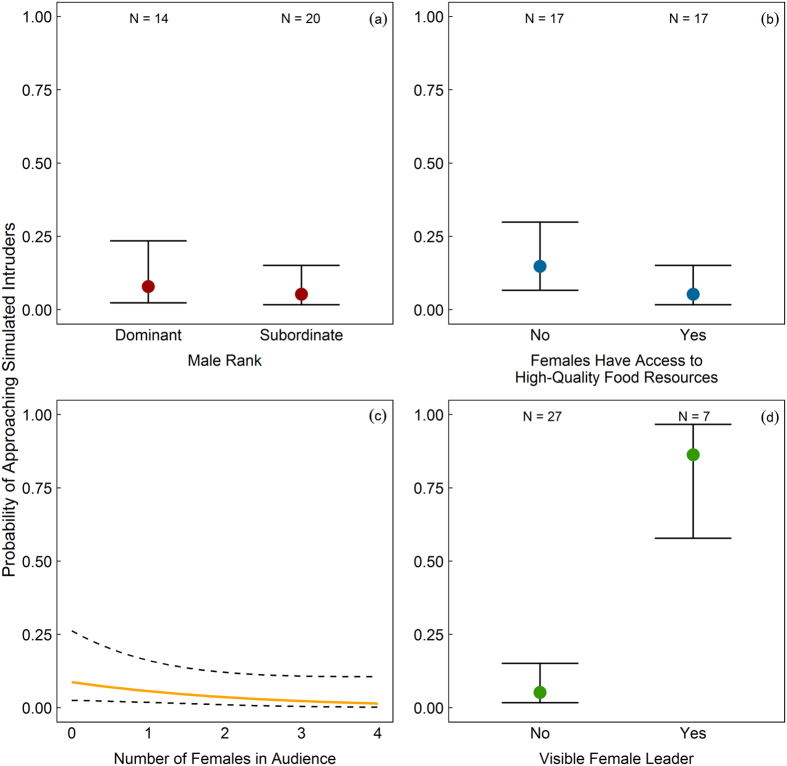
Probability that male vervet monkeys approached simulated intruders during playback experiments. Probability that males approached the speaker during playback experiments, depending on (**a**) their rank, (**b**) whether female group members did versus did not have access to provisioned resources (**c**) the number of females in their audience, and (**d**) whether or not a female group member led an approach first. Predicted values and predicted standard errors (error bars and dotted lines) were obtained by setting all additional factors in the GLMM model to their mean (or median for binary variables) value.

We found that dominant males were no more likely to approach the simulated intruders than subordinate males (GLMM: *B* = 0.44, *SE* = 1.10, *z* = 0.40, *N* = 34, *P* = 0.692; Fig. 1a). Similarly, males were no more likely to approach the speaker when females were accessing high-quality food resources than when there were no provisioning boxes present (*B* = 1.14, *SE* = 1.22, *z* = 0.94, *N* = 34, *P* = 0.349; Fig. 1b). The presence of a large female audience at the time the stimulus was played also had no significant effect on the response of focal males (*B* = −0.47, *SE* = 0.58, *z* = −0.81, *N* = 34, *P* = 0.421; Fig. 1c). However, the presence of a female leader had a strong and highly significant effect on the tendency for males to approach simulated intruders (*B* = 4.73, *SE* = 1.81, *z* = 2.62, *N* = 34, *P* = 0.009; Fig. 1d). In fact, the odds ratio (calculated from the raw data) indicates that males were 35 times more likely to approach the playback stimulus when a female group member began to vocalize and approach the speaker first. In only one of the observed cases in which a female leader was present, did the focal male ignore (rather than follow and support) her. In all cases where the focal male followed a female leader, only a single female needed to vocalize and approach the speaker before the male followed; therefore, we were unable to consider the effect that the number of female leaders had on the propensity of males to approach the simulated intruders. In only three trials did the focal male have another male in his audience, and in none of these cases did either male approach the simulated intruders. Thus, although we lacked the data for a formal investigation of the effect of male audience members, the presence of another male, or potential cooperative partner, appears to have had little effect on the response of focal males during playback experiments. Notably, there was also not a visible female leader in any of these three cases.

## Discussion

Male food defence is a seemingly puzzling behaviour given that food is not the primary resource that limits male fitness. Male food defence in vervet monkeys potentially functions to increase female reproductive success and increase male fitness indirectly, build social prestige, or improves status as a good cooperative partner. To test these three hypotheses, we employed a novel experimental approach in which we manipulated male and female access to high-quality resources and simulated an intruding group nearby. These experiments were conducted in a season when naturally occurring resources were scarce and females were gestating; thus, male food defence in this season could have a large impact on the reproductive success of female group members. However, males showed no greater tendency to engage in an intergroup encounter when females had exclusive access to high-quality food resources, than when they did not. Therefore, our results are inconsistent with the hypothesis that males defend food to improve female reproductive success in this species. This mechanism is most likely to function in territorial species where males are the philopatric sex, as these are the conditions in which the benefits of improving resource access for females could accrue over long time periods (e.g. chimpanzees^18^).

It has also been suggested that males may defend food to improve female reproductive success, even when they are the dispersing sex and their tenure in a given group is relatively short^9^. However, despite the potential indirect fitness benefits, males may fail to defend food resources on behalf of females as doing so is vulnerable to the free-rider problem^43^,^44^. In vervet monkeys for example, females are highly promiscuous, mating with multiple male group members throughout the mating season^42^; all male group members obtain at least some matings, and mating skew is often relatively low among males^19^. As a result, males who would defend food resources to indirectly increase their fitness would also provide similar benefits to their fellow male group members. Males who refrain from food defence would gain indirect fitness benefits without paying any cost, and selection would favor a defection strategy. This strategy of male food defence for indirect fitness benefits is therefore unlikely to evolve unless reproductive skew is high or males are close relatives. Neither condition applies to vervet monkeys^42^.

If male participation in intergroup conflicts functioned as a costly signal, we would expect males to show the strongest response to simulated intruders when there was a large audience of females (i.e. many potential future mates) to observe them. However, despite the fact that many of our playbacks occurred during the mating season, we found no effect of female audience size on the likelihood that males approached the speaker, suggesting that males do not use intergroup encounters as an opportunity to advertise their genetic quality to females^26^. Instead, we found that the response of males was highly dependent on the behaviour of female audience members during playback experiments. Males rarely led the approach towards simulated intruders, suggesting that it is unlikely that they were motivated by resource-based benefits. Conversely, if female group member(s) initiated an approach then males almost always followed and supported them, which supports that male participation in intergroup conflicts largely functions as intersexual cooperation. Therefore, apparent male food defence appears to be a by-product, arising from the combination of female food defence and intersexual cooperation. Previous work on our study population found that approximately a third of the variability in male mating success was related to the frequency with which males supported females in intergroup conflicts^19^, indicating there are tangible fitness benefits associated with being a cooperative group member. Future studies are necessary to determine the precise evolutionary mechanism(s) by which males build their status as a cooperative group member (e.g. direct reciprocity and/or indirect reciprocity).

Support during intergroup encounters is likely only one of many services that males provide in this species, with other potential services including predator vigilance and mobbing, coalitionary support in intragroup conflicts, tolerance around valuable food resources and grooming. Furthermore, such male services are likely more widespread among social animals than is generally appreciated, and may thus represent an important proportion of cases of male assistance in primates, other mammals, and birds. An interesting avenue of future investigation lies in understanding how the amount of choice that females are able to exert in their mating partners influences the extent and type of male services observed.

Our findings highlight that when intergroup aggression is a cooperative activity involving the joint action of multiple individuals, who an individual participates in aggressive intergroup interactions with can be more important than the resources they end up defending. Therefore, it is important to investigate both the resource-based benefits and social incentives gained from participation in intergroup aggression, and considering both the ecological and social contexts in which individuals participate is critical to doing so. Examining audience effects is a useful approach as the makeup of the audience (e.g. the number of individuals, their sex, relationships and reproductive status) as well as their activity (e.g. whether they are active or not, and who leads versus follows) can illuminate the role that social incentives play in the evolution and maintenance of cooperation^45^,^46^.

## Methods

### Field Experiments

Data were collected on three habituated groups of vervet monkeys at the Mawana Game Reserve (28°00′S, 31°12′E) in South Africa. Groups consisted of 30 to 56 individuals, with between 1 and 7 adult males and 5 and 14 adult females. We first trained individuals on the provisioning boxes between May 2012 and July 2013. The goal of training was for females to learn that the boxes contained high-quality food resources they could access, and for males to learn that because the boxes were opened only for females, they were excluded from accessing the high-quality resources within. Male exclusion ensured that males understood they would gain no direct personal benefit from defending provisioning boxes against simulated intruders so that if males did defend the provisioning boxes during playback experiments, they did not do so to increase their own food intake.

In each training session, we deployed six boxes and allowed females to access them until they lost interest. During the summer, when high-quality foods were available in the environment, females were often not interested in the boxes. During the winter, when food resources were scarce, females interacted with the provisioning boxes for 75 minutes on average. Boxes were provisioned with apple wedges embedded with two pieces of corn. All boxes were opened simultaneously to ensure that high-ranking females could not displace low-ranking females from a box. Each time the boxes were opened, researchers played a particular audible sound effect (of a musical instrument) so that group members who were out-of-sight of the provisioning boxes would be aware that females were accessing the high-quality resources (see electronic Supplementary Information for a sample video).

**Figure 2:**
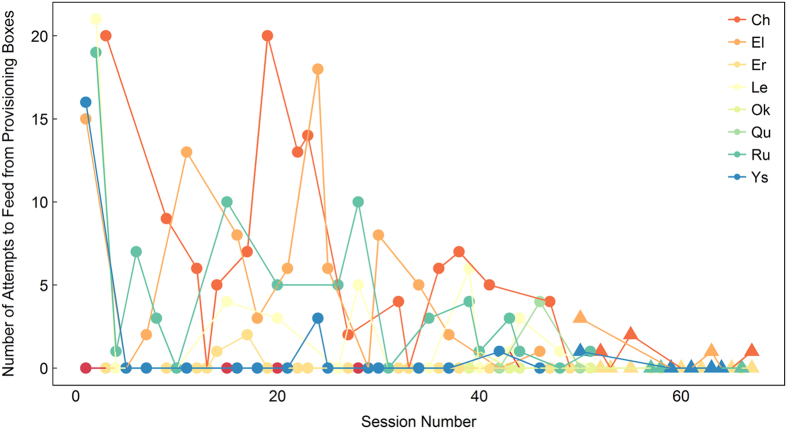
Number of attempts that each male made to access the female-only provisioning boxes. Circles represent training sessions before playback experiments commenced, triangles represent sessions after playback experiments started.

On average, males experienced 14 training sessions before the playback experiments commenced. During each training session, we recorded the number of box openings in which males attempted to gain access to the food within the provisioning boxes. When males stopped trying to open the provisioning boxes, this was taken to indicate that they had learned that they were excluded from access. Some of the males quickly learned exclusion and no longer attempted to gain access to the boxes (Fig. 2); however, other males would still test if they were able to access the boxes at the start of most training sessions. However, by the end of the training period, these males typically “gave up” and moved away from the boxes after they had observed females feeding, and saw that they were excluded, for one opening of the boxes (the maximum number of openings before giving up was 3). Once they had left (>5 m up into the tree canopy or out of sight of the provisioning boxes’ location) they never returned to attempt accessing the provisioning boxes again. Therefore, when we conducted playback trials in which females had access to high-quality resources, we always set up the provisioning boxes and allowed females to access them for ~30 minutes before conducting the playback experiment. This ensured that males who would test their access/exclusion could do so. If a male did attempt to access a provisioning box, we waited for him to move more than 5 m away from the boxes, and maintain that buffer distance for at least 10 minutes. We took this to indicate that he understood he would not gain any direct personal benefits from defending the provisioning boxes against simulated intruders.

Playback experiments were conducted in the dry season (July to November 2013) when naturally occurring fruits and seeds were scarce and females were most interested in the provisioning boxes. This period included the latter half of the mating season, the time of gestation and the beginning of the birth season. The playback stimulus consisted of a female-female conflict (recorded in a feeding context), followed 30 seconds later by a chorus contact call, which is the vocalization typically made by females during intergroup encounters. Vocalizations were recorded using a Marantz Professional solid state Recorder PMD 660 with a directional Sennheisser MKH416P48U microphone. The amplitude of stimuli was standardized (Audacity 2.0.3) so that all vocalizations sounded natural to experienced observers at 65–90 m. In each playback trial, the speaker (MiniVox Lite, Anchor Audio Ltd.) was placed so that the stimulus came from a location that was credible for the group being broadcast. The speaker was placed 65 to 90 m away from the focal individuals to ensure that the stimulus was audible but that the observer playing the stimulus was not visible. All playback experiments were conducted in areas that were used intensively by the focal group to decrease the potentially confounding effect of location^47^. In playback trials performed in conjunction with the provisioning boxes, the stimulus was played approximately 30 minutes after the boxes were first presented.

We recorded the response of one to three focal males (as well as one or more females) using handheld and time-synchronized camcorders. In each group we focal sampled both dominant and subordinate males; the number of subordinate males that were focalled varied from one to four, depending on the number of male group members and the occurrence of male immigration/emigration events. We attempted to sample focal males within each group at equal frequencies, however, this was not possible as some males immigrated/emigrated during the study period. Additionally, focal males typically needed to be near the front of the group to be within the 65 to 90 m range from the speaker, and not all males were equally likely to be in this position in the group. As a result, the number of trials per male varied from one to three in group A, one to five in group B and six to eight in group C. Focal males were usually not part of the same subgroup, and as such, were only in visible range of each other during three experimental trials (see Results section).

For each focal individual we noted the number, identity, and location (relative to the focal) of all visible group members (i.e. the audience) just prior to playing the stimulus. This ensured that when analyzing the video, we were able to determine which audience members the focal male looked at during the experiment. Once the stimulus was played back, each observer recorded the response his/her focal male, whilst speaking to the camera, noting if and when the focal moved more than 5 m from their initial location, and which visible neighbours vocalized and/or moved. The observer at the speaker also noted if and when any individuals approached the speaker. Video recordings and recorded notes from all observers present were pieced together for each trial to create a comprehensive picture of which group members vocalized and/or approached the speaker, and the exact time they did so; with these data, we were able to determine if focal males either followed, led or ignored group members.

Focal individuals were scored as approaching if their initial response was to move at least 5 m towards the speaker during the trial (i.e. within 5 minutes of the stimulus)^16^,^47^. Focal males were scored as following a female leader if this approached occurred after one or more female group members who were visible to the focal had already begun to approach the speaker (usually while vocalizing). Female vervet monkeys often initiate intergroup conflicts^19^, and to recruit support they begin to approach the opposing group while vocalizing and being vigilant. Group members who approach instigator females signal a willingness to support them and engage in an intergroup encounter. Thus, an approach was considered to indicate the willingness of the focal male to participate in an intergroup encounter.

### Statistical Analyses

We used a generalized linear mixed model (GLMM) to test if males were more likely to approach the simulated intruders when females had, versus did not have, access to high-quality food resources (provisioning boxes). We also tested the importance of male rank, the size of the female audience, and the presence of a visible female leader by including these three terms as predictor variables in the model. Because the response variable was binary, we set a binomial error structure and fit a logit link function in the model. To control for repeated observations we included the trial number, as well as the focal individual nested within group as crossed random effects^48^. We based our inferences on the full model rather than using a stepwise procedure to avoid false positives and biased effect size estimates^49^. All statistical analyses were conducted in R (version 3.0.3)^50^ using the lme4 package^51^.

### Ethical Considerations

The experimental protocol was approved by the Ezemvelo KZN Wildlife Board in South Africa and field experiments were carried out in accordance with the relevant guidelines and regulations. In addition, we took numerous steps to ensure that our experiment had a minimal effect on the behaviour of our study subjects. Because the study site is a large private game farm used for hunting (as opposed to crop farming), there was little risk that introducing the study subjects to corn in the experiment could dispose them to crop raiding. Moreover, to ensure that provisioning did not dispose the study subjects to becoming human food stealers, we trained the monkeys to only expect food in a highly specific set of conditions; if these conditions were not met, the study subjects never showed any sign that they expected humans to provide them with food. First, all researchers wore blue hats in the field. Second, on days when only observational data were collected, researchers always made “habituation” calls as they approached the study group; conversely, on days when experiments that included provisioning occurred, researchers made an alternative “food” call as they approached. Third, food was always provisioned via an experimental box (which were decorated so as to be distinct and unique), and was never given directly from a researcher to a monkey.

## Additional Information

**How to cite this article**: Arseneau-Robar, T. J. M. *et al*. Male food defence as a by-product of intersexual cooperation in a non-human primate. *Sci. Rep.* **6**, 35800; doi: 10.1038/srep35800 (2016).

## Supplementary Material

Supplementary Information

Supplementary Information
