# Supplementary material for: Male food defence as a by-product of intersexual cooperation in a non-human primate
Source: Sci Rep. 2016 Oct 24;6:35800. doi: 10.1038/srep35800 (PMC5075891; doi:10.1038/srep35800)
Supplement: Supplementary Information [file srep35800-s1.pdf]

Male food defence as a by-product of intersexual cooperation in a non-human primate

T. Jean M. Arseneau-Robar[1,2], Eliane Müller[1,2], Anouk L. Taucher[1,2], Carel P. van Schaik[1,2] and Erik P. Willems[1,2]

[1] Anthropological Institute and Museum, University of Zurich, Winterthurerstrasse 190, Zurich 8057, Switzerland

[2] Inkawu Vervet Project, Mawana Game Reserve, KwaZulu Natal, South Africa

[*] Author for correspondence (thelmajeanmarie.arseneau@uzh.ch)

Supplementary Information

Sample video demonstrating the operation of the provisioning boxes. Researchers typically deployed six of these boxes in a circle and sat in the middle; this ensured the boxes were widely spaced so that high-ranking females could not displace lower-ranking females from accessing the boxes. Researchers operated the boxes by manipulating the top string, to open the box, and the bottom string, to close and lock the box. The musical jingle ensured that all group members were aware that females were eating at the provisioning boxes, even if they were out-of-sight of the experiment location.
